# A246 BALLOON-OCCLUDED RETROGRADE TRANSVENOUS OBLITERATION FOR PRIMARY PROPHYLAXIS OF GASTRIC VARICEAL BLEEDING: A SYSTEMATIC REVIEW

**DOI:** 10.1093/jcag/gwac036.246

**Published:** 2023-03-07

**Authors:** N C Howarth, D Little, A Mujoomdar, D Cool, D Hocking, A Teriaky, K Qumosani, Q Khan, S Robert, M Brahmania, D Peck

**Affiliations:** 1 Gastroenterology, Trillium Health Partners,The Credit Valley Hospital, Mississauga; 2 Interventional Radiology; 3 Gastroenterology; 4 Radiology, London Health Sciences Centre, London; 5 Gastroenterology, Calgary Division of Gastroenterology and Hepatology, Calgary, Canada

## Abstract

**Background:**

Gastric variceal (GV) bleeding is an infrequent but serioous complication of cirrhosis that is associated with high mortality. Balloon-occluded retrograde transvenous obliteration (BRTO) is an effective treatment to prevent rebleeding from GV. The role of BRTO for primary prophylaxis of GV bleeding has not been established.

**Purpose:**

The aim of the current study is to establish the efficacy and safety of BRTO for primary prophylaxis of GV bleeding.

**Method:**

We searched EMBASE, MEDLINE, and the Cochrane Central Register of Controlled Trials from inception to November 2021. Randomized controlled trials and observational studies involving adults with cirrhosis related GV that had never bled and were treated with BRTO were included. Studies without clinical outcomes or those not reporting primary prophylaxis outcomes separately were excluded. Risk of bias was assessed using the Newcastle-Ottawa scale.

**Result(s):**

We identified 791 unique citations with 9 eligible studies after a full-text review. A total of 577 patients were included from 9 observational studies (1 prospective, 8 retrospective). Technical success after the first procedure ranged from 82.4-100% (7 studies) and GV eradication on follow-up endoscopy ranged from 88.2-100% (4 studies). GV bleeding occurred in 0-7.3% of patients with follow-up ranging from 15.8-66.2 months (9 studies). Eight studies were considered at high risk of bias due to the lack of a control group or poor comparability between the cohorts.

**Conclusion(s):**

BRTO appears to be effective for primary prophylaxis of GV bleeding. Further controlled studies are needed before BRTO can be routinely performed for primary prevention of gastric variceal bleeding.

**Please acknowledge all funding agencies by checking the applicable boxes below:**

None

**Disclosure of Interest:**

None Declared

